# Molecular characterization and evolutionary dynamics of a recombinant PDCoV strain in a swine diarrhea epidemic with SADS-CoV co-infection

**DOI:** 10.3389/fvets.2026.1749819

**Published:** 2026-02-18

**Authors:** Hong-Tao Cao, Ren-Jie Sun, Qian-Yu Qian, Dong Yang, Yang-Yang Sun, Ling-Yan Zhao, Gao-Yu Zhang, Meng-Di Zhang, Han Gu, Hong-Wei Cao, Bin Wang, Yao-Wei Huang, Yong-Le Yang

**Affiliations:** 1 Xianghu Laboratory, Hangzhou, Zhejiang, China; 2 State Key Laboratory for Animal Disease Control and Prevention, South China Agricultural University, Guangzhou, China; 3 Zhejiang Provincial Center for Animal Disease Control and Prevention, Hangzhou, China; 4 College of Life Sciences, Zhejiang Chinese Medical University, Hangzhou, China; 5 School of Pharmacy, Yancheng Teachers University, Yancheng, Jiangsu, China

**Keywords:** gut microbiota dysbiosis, PDCoV, phylogenetic analysis, SADS-CoV, viral co-infection, viral recombination

## Abstract

Porcine deltacoronavirus (PDCoV) is an emerging enteropathogenic coronavirus that poses a significant threat to the swine industry. In this study, a novel PDCoV strain, designated PDCoV-ZJHZ2024, was identified from fecal samples of diarrheic pigs in China. Metagenomic analysis revealed co-detection of PDCoV and swine acute diarrhea syndrome coronavirus (SADS-CoV), with the microbial community predominantly composed of bacteria and characterized by abnormal enrichment of *Bacillus cereus* and pronounced gut microbiota dysbiosis. Genomic analyses demonstrated that PDCoV-ZJHZ2024 has undergone independent recombination events involving the ORF1b region and the spike (S) gene, accompanied by cross-regional genetic exchange, highlighting the critical role of recombination in PDCoV evolution and diversification. Codon usage analysis further indicated that codon preferences in this strain are primarily shaped by natural selection, potentially conferring enhanced translational efficiency in the host. Collectively, these findings underscore the evolutionary adaptability and transmission potential of PDCoV-ZJHZ2024 and provide new insights into PDCoV evolutionary dynamics, thereby informing future surveillance efforts and prevention strategies in swine populations.

## Background

Animal epidemics represent a substantial threat to public health and safety, particularly in the context of current epidemic prevention and control efforts, as zoonotic transmission events may lead to serious public health consequences (1–4). Porcine diarrhea is a major health concern in swine populations, resulting in significant economic losses for the pig farming industry. Multiple viruses have been implicated in porcine diarrheal disease, including porcine epidemic diarrhea virus (PEDV) (5), porcine deltacoronavirus (PDCoV) (6), porcine circovirus type 2 (PCV2) (7), transmissible gastroenteritis virus (TGEV) (8), and swine acute diarrhea syndrome coronavirus (SADS-CoV) (9), all of which pose considerable challenges to swine health and production.

Among these, PDCoV and SADS-CoV have recently gained attention as emerging coronaviruses of concern. Although both belong to the family coronaviridae, they fall under different subgenera: SADS-CoV is classified as an Alphacoronavirus, exhibiting high genomic similarity (~95%) to bat-origin HKU2-CoV, while PDCoV is a member of the deltacoronavirus genus, with avian species considered its typical intermediate hosts (10, 11). Notably, PDCoV has been detected in human cases, and its spike (S) protein facilitates cross-species transmission through binding to the aminopeptidase N (APN) receptor (12–14). SADS-CoV also demonstrates broad host tropism, having been shown to infect human primary cells and diverse mammalian cell lines (2, 15). These viruses can cause high pre-weaning mortality rates-reportedly up to 90%-alongside severe economic losses. Their zoonotic potential further intensifies public health risks and complicates animal disease control, posing dual threats to both veterinary and human health systems (16, 17). Coronaviruses are characterized by high genetic plasticity, largely due to the lack of proofreading function in RNA-dependent RNA polymerase, as well as frequent recombination events that may facilitate host switching and viral adaptation. Such genetic features contribute to the unpredictability of emerging infectious diseases and underscore the persistent threat posed by novel coronavirus outbreaks (18).

The etiological complexity of porcine diarrhea, often involving synergistic viral co-infections, poses additional challenges to effective disease surveillance and control (19). Viruses such as PEDV, PDCoV, TGEV and SADS-CoV are frequently detected in co-infections (20–25), which may interact to enhance pathogenicity, exacerbate intestinal damage, and complicate clinical presentation (4, 26–30). Accumulating evidence suggests that co-infections significantly influence viral replication dynamics, tissue tropism, and host immune responses. For example, co-infection with PCV2 and porcine parvovirus (PPV) can induce elevated expression of intestinal cytokines, intensifying inflammatory responses and revealing strain-dependent differences in immune modulation (20). In the respiratory context, co-infection with porcine parvovirus (PorPV) and swine-origin H1N1 influenza virus has been shown to aggravate pulmonary lesions, suggesting that persistent PorPV infection may influence subsequent viral pathogenesis (21). Among enteric coronaviruses, co-infection with PDCoV and PEDV not only intensifies clinical symptoms and prolongs viral shedding but also exacerbates mucosal damage and upregulates pro-inflammatory cytokine expression, demonstrating a pronounced synergistic effect (22, 24). Large-scale surveillance studies have further revealed high frequencies of dual or triple infections involving PCV2, PCV3, and PEDV, potentially driven by genotypic diversity and amino acid mutations (23). Collectively, viral co-infections are associated with heightened disease severity, immune dysregulation, and may facilitate adaptive viral evolution, underscoring the importance of incorporating co-infection dynamics into disease management strategies (31, 32).

In this study, diarrheal samples from a pig farm in China underwent preliminary screening, with detection of SADS-CoV N protein. Given the complexity of clinical symptoms, qPCR and high-throughput sequencing were employed to systematically detect multiple viral targets and comprehensively characterize the pathogen spectrum and co-infection patterns. PDCoV was identified as the primary causative agent, while SADS-CoV was detected at low levels, indicating a latent infection. This investigation aimed to elucidate the viral composition of diarrheal samples and explore the mutation, recombination, and co-infection mechanisms associated with a novel PDCoV strain. By characterizing its genetic variation and evolutionary dynamics, the study provides a scientific foundation for vaccine development, diagnostic optimization, and targeted disease control strategies, thereby contributing to improved management of porcine diarrhea and the sustainable development of the swine industry.

## Materials and methods

### Clinical sample collection and preparation

Three diarrheal samples were received by the laboratory from pigs exhibiting clinical signs of diarrhea at a pig farm in Zhejiang Province, China. Samples were collected using sterile sampling tools, immediately placed in 5 mL of viral transport medium, labeled, and transported on dry ice to the laboratory for further processing. Upon arrival at the laboratory, 800 μL of sterile 1 × PBS was added to the centrifuge tubes containing the fecal samples. Cotton swabs were agitated using a pipette tip, followed by vigorous vortexing for 2 min. The supernatant was transferred to a new centrifuge tube, followed by centrifugation at 4 °C, 8000 rpm for 2 min. The supernatant was transferred into new tubes, labeled, and stored at −80 °C. All sampling procedures were conducted with the consent of the farm owners and in accordance with institutional animal welfare guidelines.

### Extraction of viral RNA from clinical samples

The Viral RNA was extracted from processed fecal supernatants using the Trizol-based method. A 250 μL aliquot of the clarified supernatant was transferred to a 1.5 mL RNase-free microcentrifuge tube, followed by the addition of 1 mL Trizol reagent. The mixture was incubated on ice for 5 min, after which 200 μL chloroform was added. Following a 2-min incubation at room temperature, the sample was centrifuged at 12,000 rpm for 15 min at 4 °C. The aqueous phase (approximately 550 μL) was carefully transferred to a new tube and mixed with an equal volume of pre-chilled isopropanol. The mixture was incubated at −20 °C for 60 min and subsequently centrifuged at 12,000 rpm for 15 min at 4 °C. The resulting RNA pellet was washed once with 1 mL of ice-cold 75% ethanol and centrifuged at 12,000 rpm for 5 min at 4 °C. After removal of the supernatant, the pellet was air-dried and resuspended in 20 μL of DEPC-treated water. Purified RNA samples were stored at −80 °C until further use.

### Quantitative detection of PDCoV, SADS-CoV, PEDV, and TGEV by real-time PCR targeting N genes

The viral RNA was extracted using the Trizol method and reverse transcribed into complementary DNA (cDNA) using the PrimeScript™ RT Reagent Kit (Takara, Japan), according to the manufacturer’s instructions. Quantitative real-time PCR (qPCR) was performed to detect the N genes of PDCoV, TGEV, PEDV, and SADS-CoV. Primer and probe sequences used for RT-qPCR are listed in Supplementary Table S1. Each 20 μL reaction contained TaqMan Fast Universal PCR Master Mix, specific primers and probes, and nuclease-free water. Thermal cycling was conducted under the following conditions: 50 °C for 30 min, 95 °C for 10 min, followed by 40 cycles of 95 °C for 15 s and 60 °C for 1 min. Samples with a cycle threshold (Ct) value ≤35 were considered positive.

### Synthesis of single-stranded cDNA and PCR detection

The total RNA was extracted from fecal samples using Trizol reagent (Life Technologies, USA), and reverse transcription was performed using the PrimeScript™ RT Reagent Kit (Thermo Fisher, USA) to generate complementary DNA (cDNA). PCR amplification was conducted in a 20 μL reaction mixture containing 10 μL of 2 × Taq PCR Master Mix, 0.5 μL each of forward and reverse primers (10 μM), 2 μL of cDNA template, and RNase-free water. The thermal cycling protocol included an initial denaturation at 95 °C for 5 min, followed by 35 cycles of 95 °C for 30 s, 55 °C for 30 s, and 72 °C for 1 min, with a final extension at 72 °C for 5 min. PCR products were separated by electrophoresis on a 1.5% agarose gel and visualized under UV illumination.

### Whole-genome sequencing of porcine clinical samples

The clinical fecal samples were collected from pig farms and subjected to standardized preprocessing procedures, including dehydration, homogenization, and filtration, to ensure sample purity and consistency. The Total RNA was extracted using commercially available RNA extraction kits, and RNA concentration and purity were assessed to confirm suitability for downstream applications. Depending on the experimental design, either mRNA-enriched or total RNA libraries were prepared. Library construction included reverse transcription to synthesize complementary DNA (cDNA), PCR amplification, and quality control of the final libraries. Sequencing was performed on the MGI high-throughput sequencing platform (e.g., MGISEQ or DNBSEQ series), generating whole-genome sequencing data. Raw reads were subjected to rigorous quality control procedures to remove low-quality sequences, and only high-quality reads were retained for subsequent analyses. Bioinformatic processing included sequence alignment and functional annotation using appropriate computational tools. These analyses enabled comprehensive microbial diversity profiling and the identification of potential functional genes and microbial biomarkers. A detailed analytical report was generated based on the results, and all data were securely archived in a dedicated database for future access and research applications (33).

### Phylogenetic inference and genomic characterization of PDCoV

A total of 193 complete PDCoV genome sequences were retrieved from the NCBI database. To assess the evolutionary relationships and recombination-associated phylogenetic incongruence, phylogenetic analyses were performed on seven datasets, including the complete genome, full-length S gene, ORF1b gene, and recombination breakpoint-defined regions of the genome and S gene. Multiple sequence alignments were conducted using MAFFT implemented in BioAider v1.727, and maximum likelihood phylogenetic trees were inferred using IQ-TREE v3.0.1 with 1,000 bootstrap replicates. The best-fit nucleotide substitution models were selected based on the Bayesian Information Criterion. All trees were visualized using iTOL (34).

### Codon usage analysis of the PDCoV genome

The complete genome sequences of PDCoV, encompassing all open reading frames and coding regions, were retrieved from the GenBank database. Multiple sequence alignment was performed using MUSCLE (Windows 64) to ensure comparative analysis within a consistent reference framework. Codon usage analysis was subsequently conducted using CodonW software, and the Relative Synonymous Codon Usage (RSCU) values were calculated to evaluate codon preference across the viral genome. Codon usage patterns were visualized using an online tool (http://112.86.217.82:9929/), which generated bar charts and heatmaps illustrating codon usage frequencies in different genes or genomic regions. In addition to RSCU values, the Effective Number of Codons (ENC) was calculated to quantify overall codon usage bias, and the GC content at the third codon position (GC_3_) was determined to provide further insight into compositional constraints influencing codon selection. To comprehensively assess codon usage bias, ENC-GC_3_ plots (ENC plots) were generated using the Genepioneer online platform. These plots, which depict the relationship between ENC values and GC_3_ content, enabled evaluation of the extent to which codon usage is influenced by mutational pressure versus natural selection. This integrated analytical framework provided a systematic understanding of codon usage characteristics in the PDCoV genome and their potential implications for viral gene expression and evolutionary adaptation.

### Recombination analysis of the PDCoV genome

The recombination events in the PDCoV genome were systematically analyzed using RDP4 (Recombination Detection Program, version 4) (35–37). Multiple sequence alignments of the full-length PDCoV genomes and spike (S) gene sequences were performed using the MAFFT algorithm implemented in BioAider v1.727. The aligned datasets were subsequently imported into RDP4 for recombination detection. Recombination analyses were conducted using multiple algorithms, including RDP, BootScan, MaxChi, and GENECONV, with default parameters applied. Recombination breakpoints and putative parental sequences were inferred by RDP4 based on consensus results from multiple methods. Only recombination events supported by supported by two or more independent algorithms and meeting the statistical significance threshold of *p* < 0.05 were considered reliable and retained for further analysis (Table 1).

**Table 1:** Predicted recombination events in complete PDCoV genomes.

Event number (No.)	Found in	Recomb.	Major parent	Minor parent	Detection methods						
					R	G	B	M	C	S	T
1	2	OQ736716	MN025260	MF280390	**−**	**−**	**+**	**+**	**−**	**+**	**+**
2	2	OK649355	OR122654	MN520196	**+**	**+**	**+**	**+**	**+**	**+**	**+**
3	1	MN173781	MZ388471	MN173779	**−**	**+**	**−**	**+**	**+**	**+**	**+**
4	3	MN520196	MH715491	ON402372	**+**	**+**	**−**	**+**	**+**	**+**	**+**
5	3	MH118333	KX361344	KR265859	**+**	**+**	**+**	**+**	**+**	**+**	**+**
6	1	KX443143	KX361344	KR265864	**−**	**−**	**−**	**+**	**+**	**+**	**+**
7	22	KT266822	KX361343	KT336560	**−**	**+**	**−**	**+**	**+**	**+**	**+**
8	8	KU981059	MK625641	KU665558	**−**	**+**	**+**	**+**	**+**	**−**	**+**
9	1	MN781985	OQ547740	KX361344	**−**	**+**	**+**	**+**	**+**	**−**	**+**
10	1	MK355396	MK572803	MK330604	**−**	**+**	**+**	**+**	**+**	**+**	**+**
11	1	MK330604	MK572803	KX998969	**−**	**+**	**−**	**+**	**+**	**+**	**+**
**12**	**10**	**ON968724**	**PDCoV-ZJHZ2024**	**OM256446**	**−**	**+**	**+**	**+**	**+**	**+**	**+**
13	2	MZ388472	MH700628	MZ388470	**+**	**+**	**+**	**+**	**+**	**+**	**−**
14	1	MF642325	MF642324	MF642322	**−**	**−**	**−**	**+**	**+**	**+**	**+**
15	9	MN520200	MN025620	KP757891	**+**	**−**	**−**	**+**	**+**	**+**	**+**
16	15	OK546242	KX361344	MF095123	**−**	**+**	**−**	**+**	**+**	**+**	**+**
17	6	MZ388473	MK993519	MZ388471	**−**	**+**	**−**	**+**	**+**	**+**	**+**
18	5	KY065120	MF280390	KT266822	**−**	**−**	**−**	**+**	**+**	**+**	**+**
19	1	KT021234	KU665558	MZ802774	**+**	**+**	**−**	**+**	**+**	**+**	**+**
20	69	KX361344	KJ584357	MF642324	**−**	**−**	**+**	**+**	**+**	**+**	**+**
21	1	KT021234	MH715491	MN781985	**−**	**+**	**−**	**+**	**+**	**+**	**+**
22	5	OQ551110	MW854634	MN520206	**+**	**+**	**+**	**+**	**+**	**+**	**+**
23	3	MF642323	KP757890	MW685623	**−**	**−**	**+**	**+**	**+**	**+**	**+**
24	2	OP792038	OR122654	MF095123	**+**	**−**	**−**	**−**	**−**	**−**	**−**
25	3	MW816149	MN520209	ON402372	**−**	**−**	**−**	**+**	**−**	**+**	**+**
26	62	MF280390	MH715491	OQ551110	**−**	**−**	**−**	**+**	**+**	**+**	**+**
27	33	MW685623	MN520198	KY354363	**−**	**−**	**−**	**+**	**+**	**+**	**+**
28	2	KX022605	KX022603	MN520198	**−**	**−**	**−**	**+**	**−**	**−**	**+**
29	2	KU665558	MH025764	KJ584357	**−**	**−**	**−**	**+**	**+**	**+**	**−**
30	7	KR131621	MH025763	KY513725	**−**	**−**	**−**	**+**	**−**	**−**	**+**
31	1	MT663769	MH025762	KP757890	**−**	**−**	**−**	**+**	**−**	**−**	**+**
32	38	OQ566228	MT663769	MT227371	**−**	**+**	**−**	**−**	**−**	**−**	**−**
33	3	MN520198	KJ584356	KR265863	**−**	**−**	**−**	**−**	**−**	**+**	**+**
34	1	MF642325	KP757890	KX998969	**−**	**−**	**−**	**+**	**+**	**+**	**−**
35	7	MZ388469	KU051649	MT227371	**−**	**−**	**−**	**−**	**−**	**−**	**+**

*Algorithms used for recombination detection: RDP(R), GENECONV(G), BootScan(B), MaxChi(M), Chimaera(C), SiScan(S), TOPAL DSS(T).

Bold text indicates the recombination event identified in the PDCoV-ZJHZ2024 strain in this study.

## Results

### Detection of PDCoV and SADS-CoV with concurrent gut dysbiosis in this swine diarrheal case

**Figure 1:**
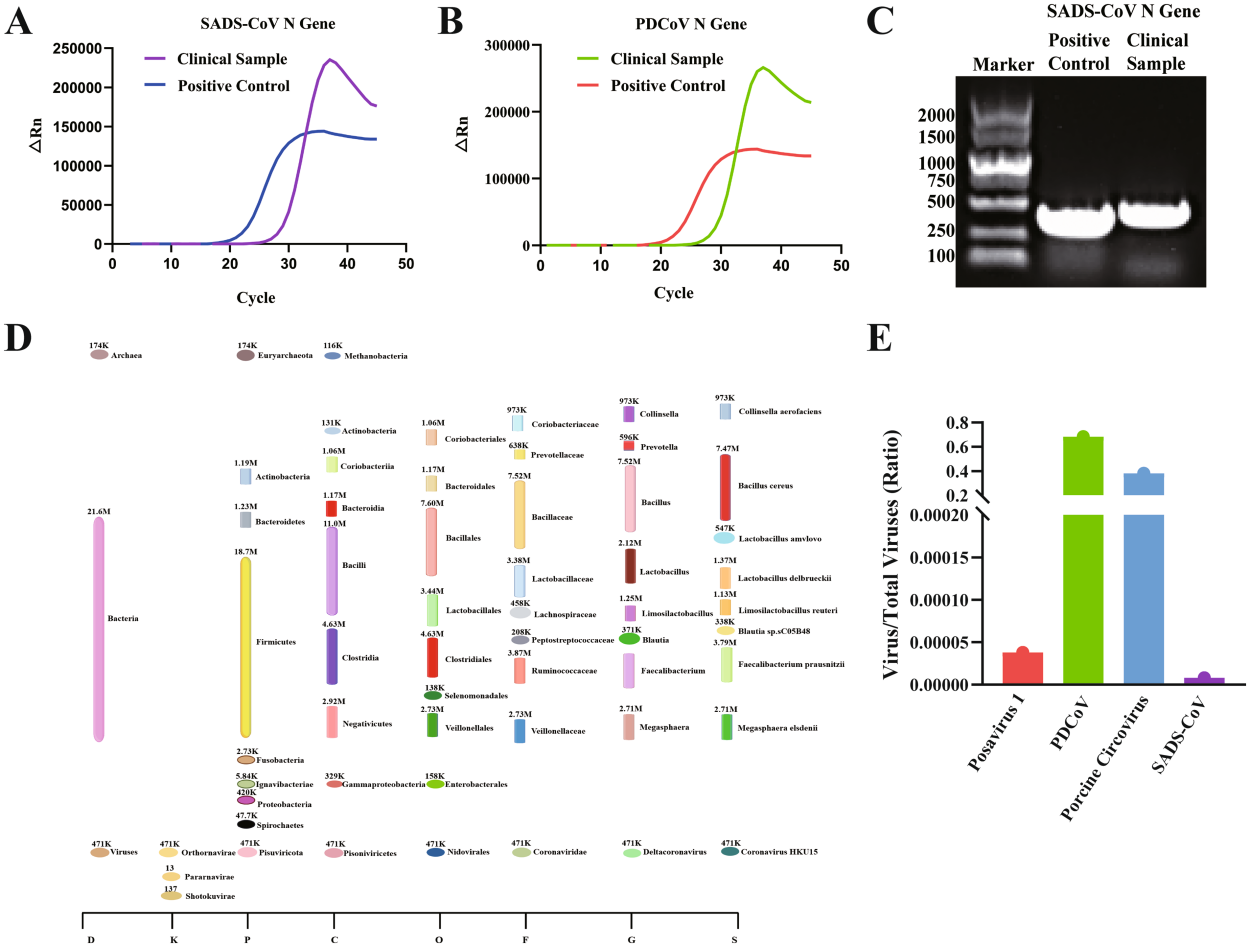
Molecular and metagenomic identification of pathogens associated with swine diarrhea. **(A)** Quantitative PCR (qPCR) detection of SADS-CoV N gene in fecal samples collected from diarrheic pigs. Amplification curves indicate positive signals in both the sample and positive control. **(B)** qPCR detection of the PDCoV N gene, showing a clear amplification curve with a cycle threshold (Ct) value below 35, confirming the presence of PDCoV in the samples. **(C)** Agarose gel electrophoresis of PCR products targeting the N gene of PDCoV, PEDV, TGEV, and SADS-CoV. A specific band of approximately 450 bp confirms the presence of SADS-CoV. Only PDCoV and SADS-CoV were detected, whereas PEDV and TGEV were negative. **(D)** Taxonomic composition of the fecal microbial communities based on high-throughput metagenomic sequencing. Bacteria were the predominant domain, followed by viruses and archaea. Within the virome, PDCoV accounted for the largest proportion (68%), followed by porcine circovirus (PCV, 31%), and a minor proportion of Posavirus 1. SADS-CoV was not detected at the metagenomic level. This figure was created using Figdraw (www.figdraw.com). **(E)** Relative abundance of specific viruses within the total viral community based on whole-genome metagenomic data. PDCoV exhibited the highest abundance, enabling the successful assembly of a novel strain, PDCoV-ZJHZ2024, which was subjected to further genomic and evolutionary analyses.

This study performed comprehensive molecular and metagenomic analyses on clinical fecal samples collected from diarrheic pigs at a pig farm in Zhejiang Province, China, with the aim of identifying the principal causative agents and associated alterations in gut microbial composition during the outbreak. The qPCR revealed the presence of both PDCoV and SADS-CoV, while PEDV and TGEV tested negative (Figures 1A,B). The detection of SADS-CoV was further validated by agarose gel electrophoresis and sequencing of its N gene fragment (Figure 1C and Supplementary Figure S2A). High-throughput sequencing indicated that the microbial community was predominantly composed of bacteria, followed by viruses and a minor proportion of archaea (Figure 1D).

Within the virome, PDCoV accounted for the highest relative abundance (68%), followed by porcine circovirus (PCV) at 31%, with Posavirus 1 detected at low levels (Figure 1E). Although SADS-CoV was not identified by metagenomic sequencing, subsequent qPCR analysis detected partial genomic fragments, including ORF1a and the spike (S) gene regions. Due to the low viral load, *de novo* assembly of the complete SADS-CoV genome was not feasible (Supplementary Figure S1 and Supplementary Figure S2B), suggesting the virus may persist in a latent or low-level infection state, warranting continued surveillance. Further analysis of the sequencing data led to the successful identification and assembly of a novel PDCoV strain, designated PDCoV-ZJHZ2024. The complete genome sequence of PDCoV-ZJHZ2024 generated in this study has been deposited in GenBank under the accession number PX492333.

Metagenomic profiling also revealed pronounced dysbiosis in the gut microbiota of the affected pigs. The total bacterial abundance reached 21.6 million reads, with Firmicutes (18.7 M) markedly predominant over Bacteroidetes (1.23 M), resulting in a severely imbalanced Firmicutes/Bacteroidetes ratio. Members of the Bacilli (11.0 M) and Clostridia (4.63 M) classes were significantly enriched. Notably, the conditional pathogen *Bacillus cereus* was detected at a high abundance (7.47 M), indicating a potential contributory role in the pathogenesis of the outbreak. Although beneficial microbes such as *Faecalibacterium prausnitzii*, *Lactobacillus* spp., and *Megasphaera elsdenii* were present, their relative abundances were substantially reduced, suggesting a loss of ecological competitiveness and compromised intestinal barrier function. The dominance of PDCoV (Coronavirus HKU15) supports its role as the primary pathogen and further implies that viral infection may disrupt intestinal homeostasis, thereby promoting the proliferation of opportunistic bacteria and exacerbating disease severity (Figure 1D).

In summary, PDCoV was identified as the primary etiological agent responsible for this diarrheal outbreak, while SADS-CoV was present at low levels, indicative of latent infection. The pronounced dysbiosis of the gut microbiota—characterized by depletion of beneficial bacteria and expansion of conditional pathogens—was likely triggered by viral infection and may have contributed synergistically to disease progression.

### Molecular evolution and phylogenetic characteristics of PDCoV-ZJHZ2024

Nucleotide BLAST analysis showed that the newly identified PDCoV strain PDCoV-ZJHZ2024 shared 98.38–99.63% nucleotide identity with previously reported PDCoV strains, with PDCoV-CHLNFX2022 being its closest relative. Comparative genomic analysis between PDCoV-ZJHZ2024 and PDCoV-CHLNFX2022 identified more than 60 nucleotide substitutions distributed across both coding and non-coding regions of the genome (Supplementary Table S2). Notably, a relatively high proportion of nonsynonymous substitutions was concentrated in the spike (S) gene, with approximately 40 amino acid variations detected, suggesting that this region may be subject to strong evolutionary selective pressure.

Several amino acid substitutions were identified in the S protein, including H19751Y, H19753Y, P20255L, and I20385T. These mutations may influence the structural properties or antigenic epitopes of the spike protein, thereby potentially affecting viral entry and host immune recognition.

Within the ORF1ab region, multiple nonsynonymous substitutions were distributed in functional domains associated with viral replication, which may affect viral replication efficiency. Overall, PDCoV-ZJHZ2024 exhibits substantial genetic diversity across multiple genomic regions, reflecting its dynamic evolutionary characteristics. The potential functional consequences of these mutations warrant further experimental investigation.

### Phylogenetic incongruence and evidence of inter-provincial genetic exchange

**Figure 2:**
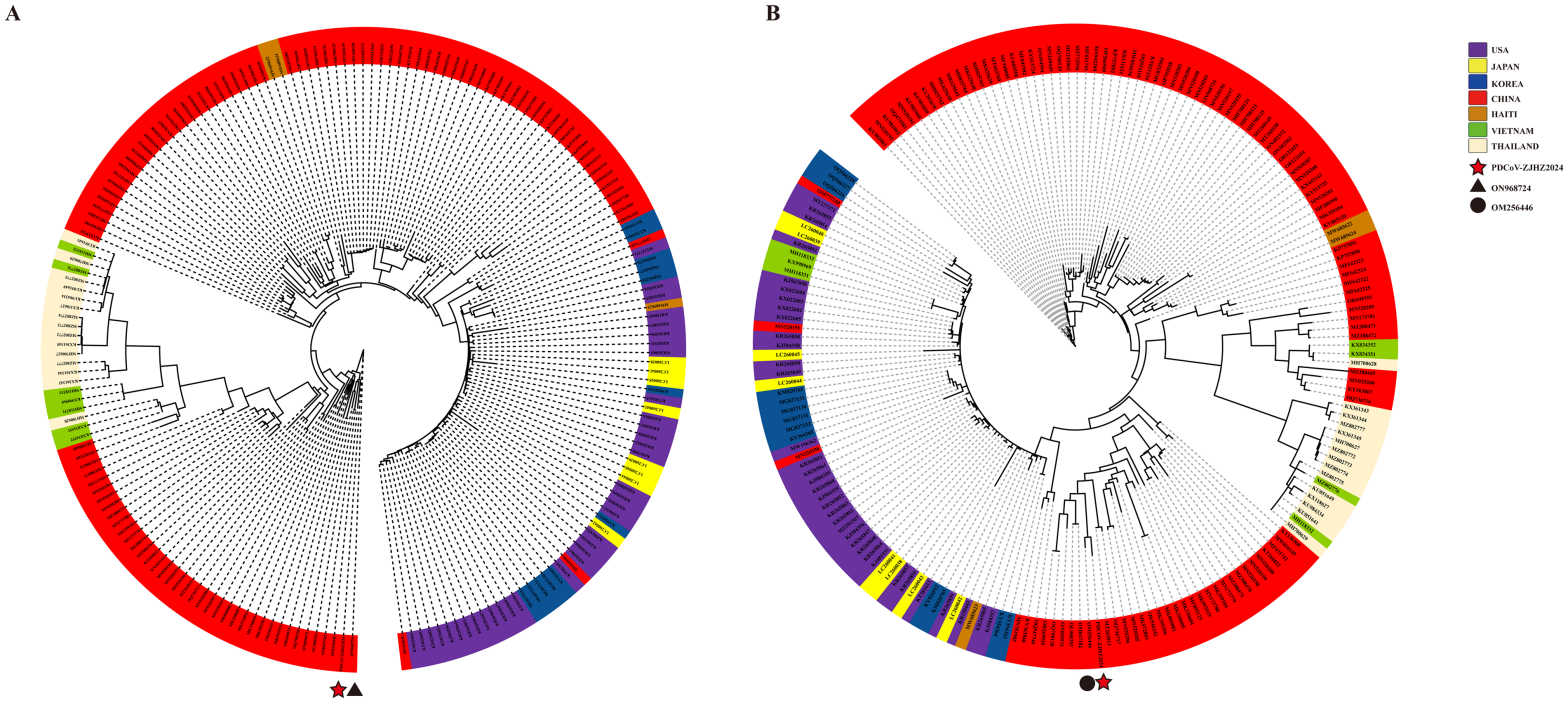
Phylogenetic analysis of the novel PDCoV-ZJHZ2024 strain. **(A)** Maximum likelihood phylogenetic tree constructed using 194 full-length PDCoV genome sequences. The analysis delineates three major evolutionary lineages: the China lineage, the USA lineage, and the Southeast Asia lineage (including strains from Thailand, Vietnam, etc.). The novel strain PDCoV-ZJHZ2024 clusters within the China lineage and exhibits high genetic similarity to strain CHLNFX2022 (GenBank: ON968724). **(B)** Phylogenetic tree based on the spike (S) gene sequences of PDCoV strains. The topology differs from the whole-genome tree, with PDCoV-ZJHZ2024 showing closer clustering with strain CH-SDLY52-2021 (GenBank: OM256446), suggesting potential recombination or region-specific adaptation in the S gene. Color legends: Country origins of strains are color-coded as follows: USA (purple), Japan (yellow), Korea (blue), China (red), Haiti (green), Vietnam (dark green), and Thailand (beige). Strain-specific markers: PDCoV-ZJHZ2024 (★), CHLNFX2022 (▲), CH-SDLY52-2021 (●).

Using 193 reference sequences retrieved from the NCBI database, full-genome and spike (S) gene phylogenetic analyses were performed for PDCoV-ZJHZ2024 (Figures 2A,B). The full-genome phylogenetic tree revealed that PDCoV strains segregate into three major lineages: the Chinese lineage, the American lineage, and the Southeast Asian lineage. The Chinese lineage primarily comprises strains isolated within China, whereas the American lineage mainly includes strains from the United States, Peru, Japan, and South Korea. The Southeast Asian lineage consists of strains from Thailand, Vietnam, and Laos, exhibiting considerable genetic and geographic diversity.

Notably, several strains from South Korea and Japan clustered within the American lineage, suggesting possible introductions from North America. In addition, two strains from China (MN520191 and MN520198) were also grouped within the American lineage, indicating that American-lineage PDCoV strains may have been introduced into China through transboundary transmission.

In the full-genome phylogenetic analysis, PDCoV-ZJHZ2024 clustered within the Chinese lineage and showed the closest phylogenetic relationship to PDCoV-CHLNFX2022 (ON968724), suggesting a potential epidemiological linkage between the two strains. However, phylogenetic analysis based on the S gene (Figure 2B) revealed a partially incongruent clustering pattern compared with the full-genome tree. Specifically, the S gene of PDCoV-ZJHZ2024 showed the highest sequence similarity to PDCoV-CH-SDLY52-2021 (OM256446) rather than PDCoV-CHLNFX2022. This topological incongruence between the full-genome and S gene phylogenies suggests that a recombination event involving the spike gene may have occurred, a phenomenon commonly observed in coronaviruses, and this inference was further supported by subsequent recombination analyses.

Further comparison of the phylogenetic positions of PDCoV-ZJHZ2024 across different genomic regions indicated region-specific divergence. The full-genome phylogeny suggested a closer relationship to strains from Liaoning Province, whereas the S gene phylogeny showed greater similarity to strains from Shandong Province. This pattern supports the occurrence of inter-provincial genetic exchange among PDCoV strains circulating in China.

### Codon usage patterns in PDCoV-ZJHZ2024 reveal translation optimization dominated by natural selection

Codon usage bias in the newly sequenced PDCoV strain PDCoV-ZJHZ2024 was analyzed based on relative synonymous codon usage (RSCU) patterns and the distribution of the effective number of codons (ENC). RSCU analysis evaluates deviations between the observed frequencies of synonymous codons and their expected frequencies under equal usage assumptions, thereby reflecting codon usage preferences and potential evolutionary constraints within the viral genome.

**Figure 3:**
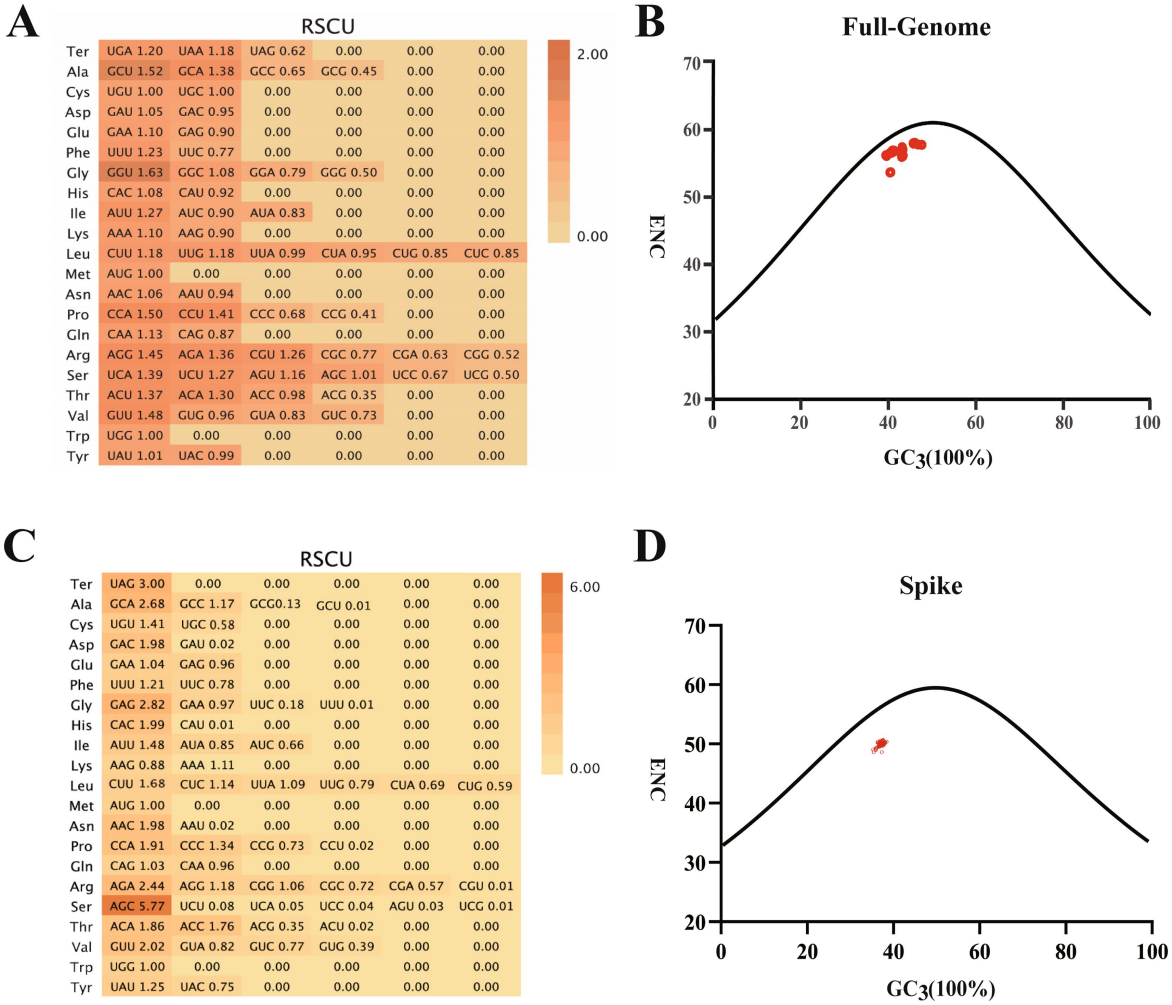
Codon usage bias analysis of the PDCoV complete genome and spike (S) gene. **(A)** Relative synonymous codon usage (RSCU) values calculated for the full-length PDCoV genome. Codons with RSCU > 1 are preferentially used, whereas those with RSCU < 1 are underrepresented. The analysis reveals a distinct codon usage bias, suggesting adaptation of the virus to host-specific translational mechanisms. **(B)** ENC-GC_3_ plot of the PDCoV complete genome, depicting the relationship between the effective number of codons (ENC) and the GC content at the third codon position (GC_3_). Most data points lie below the expected curve, indicating that codon usage is influenced not only by mutational bias but also by natural selection. **(C)** RSCU analysis of the spike (S) gene. The S gene displays a more pronounced codon usage bias than the full genome, with several codons significantly overrepresented. This pattern may reflect selection for efficient protein translation or structural-functional constraints related to viral entry. **(D)** ENC-GC_3_ plot of the S gene. Similar to the full genome, the codon usage of the S gene deviates from the neutral expectation, suggesting the combined influence of mutation pressure and translational selection on its evolution.

The RSCU results showed that GCU, GGU, and CCA were the most frequently used codons across the complete genome (Figure 3A). In contrast, the S gene exhibited preferential usage of codons such as UAG, GCA, GAG, AGA, AGC, and GUU (Figure 3C), indicating distinct codon usage patterns among different genomic regions.

Furthermore, ENC–GC3 plot analysis revealed that data points from both the complete genome and the S gene of PDCoV-ZJHZ2024 deviated from the theoretical curve expected under mutation pressure alone (Figures 3B,D), suggesting that codon usage bias may be influenced by natural selection. These results indicate that, in addition to mutational pressure, evolutionary selection factors may play a role in shaping codon usage patterns in PDCoV.

### Evolutionary dynamics and parental origins of PDCoV-ZJHZ2024 inferred from full-genome and spike gene recombination

**Figure 4:**
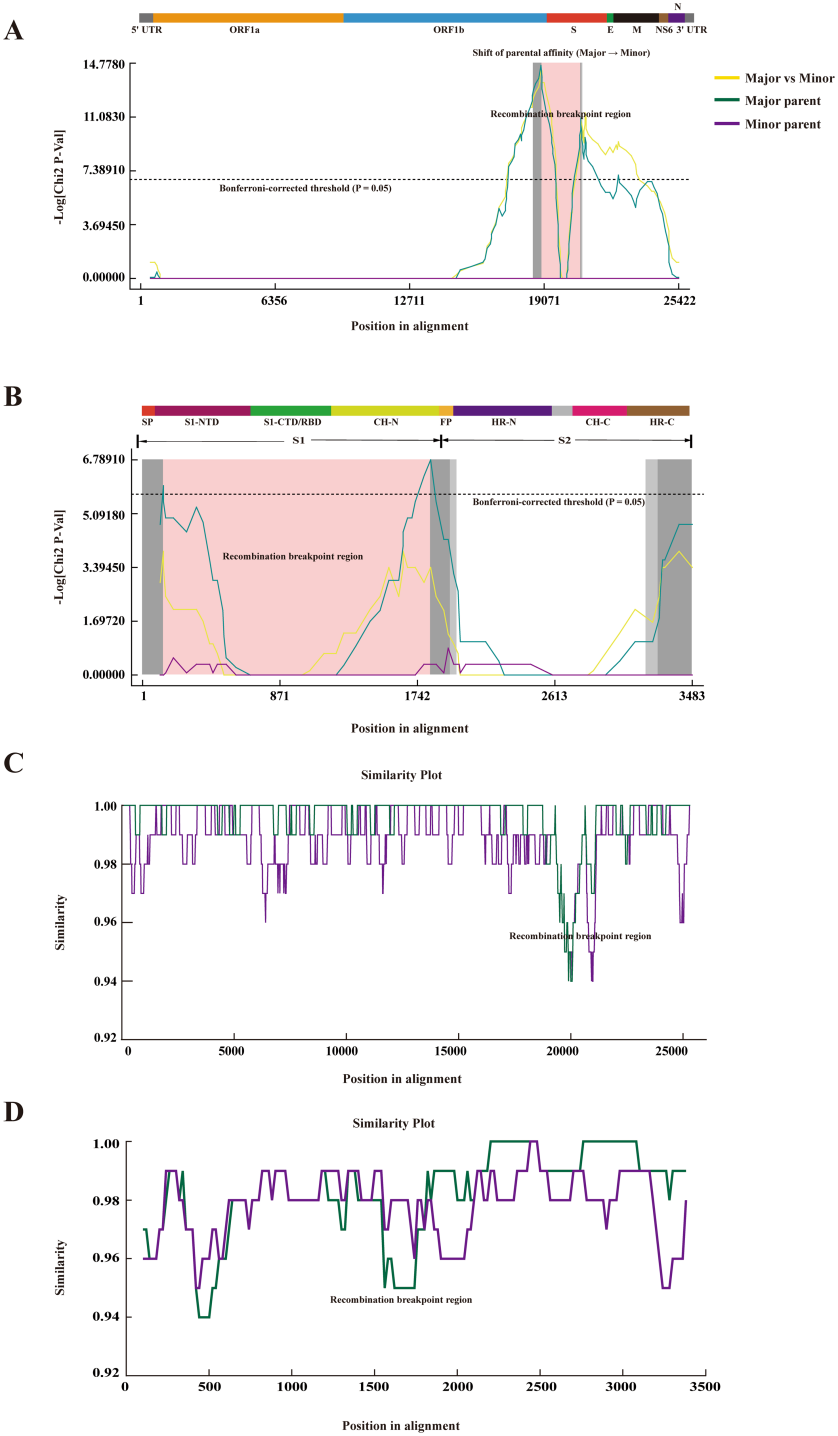
Cross-validation of a recombination event in PDCoV-ZJHZ2024 using different analytical methods. **(A)** Whole-genome recombination detection of PDCoV-ZJHZ2024 based on the MaxChi method. The *x*-axis indicates nucleotide positions in the aligned genome, and the *y*-axis represents −log₁₀(*p*-value). The dashed line denotes the Bonferroni-corrected significance threshold (*p* = 0.05). Colored curves represent the major parent, minor parent, and their differential signal. The shaded gray region indicates the inferred recombination breakpoint region, located at the ORF1b–S gene junction. A schematic representation of the PDCoV genome organization is shown above. **(B)** Recombination analysis within the S gene of PDCoV-ZJHZ2024 using the MaxChi method. The *x*-axis represents nucleotide positions in the aligned S gene, and the *y*-axis shows −log₁₀(*p*-value). The dashed line indicates the significance threshold (*p* = 0.05). The pink shaded region highlights the recombination breakpoint detected within the S gene. A schematic diagram of the S protein domains (S1, S2, and functional subdomains) is shown above. **(C)** SimPlot similarity analysis at the whole-genome level. PDCoV-ZJHZ2024 was used as the query sequence to assess sequence similarity with the inferred major and minor parental strains. The *x*-axis indicates genomic position and the *y*-axis represents nucleotide similarity. The arrow marks the recombination breakpoint region consistent with the MaxChi analysis. **(D)** SimPlot similarity analysis of the S gene. Changes in sequence similarity between PDCoV-ZJHZ2024 and the major and minor parental strains across different regions of the S gene are shown, further supporting the presence of an independent recombination event within the S gene.

Systematic recombination analyses revealed that PDCoV-ZJHZ2024 possesses a clear mosaic genomic architecture, indicating that its evolutionary trajectory has been strongly shaped by recombination events. At the whole-genome level, multiple algorithms implemented in the RDP package (GENECONV, BootScan, MaxChi, Chimaera, SiScan, and 3Seq) consistently detected a highly significant recombination event (Event No. 12; Table 2). This recombinant fragment was located at the ORF1b–S junction region, spanning alignment positions 18,958–20,798 (18,941–20,778 after gap removal), with both breakpoints supported by narrow 99% confidence intervals. The extremely low *p-*values obtained across methods (global KA, *p* = 1.39 × 10^−19^) further confirmed the robustness of this event. Consistently, MaxChi analysis revealed a clear parental lineage switch within the ORF1b–S region, with −log₁₀(*p*-values) exceeding the Bonferroni-corrected threshold (Figure 4A and Supplementary Figure S4A), while SimPlot analysis showed an abrupt shift in nucleotide similarity between PDCoV-ZJHZ2024 and the putative parental strains across the same region (Figure 4C and Supplementary Figure S4C), jointly supporting a reliable whole-genome-scale recombination event.

**Table 2:** Confidence analysis of recombination events in PDCoV whole genome and S gene.

Recombination Event Number	Methods	#seqs detected in	Av. *P*-val
Confidence analysis of whole-genome recombination event 16 in PDCoV	RDP	--	--
	GENECONV	10	2.771 × 10^−3^
	BootScan	4	1.577 × 10^−6^
	MaxChi	10	2.011 × 10^−5^
	Chimaera	10	7.816 × 10^−5^
	SiScan	3	7.899 × 10^−9^
	3Seq	1	3.790 × 10^−11^
	Lard	--	--
	Phylpro	--	--
Confidence analysis of S-genome recombination event 4 in PDCoV	RDP	--	--
	GENECONV	--	--
	BootScan	--	--
	MaxChi	1	4.030 × 10^−3^
	Chimaera	1	3.148 × 10^−2^
	SiScan	1	2.910 × 10^−5^
	3Seq	--	--
	Lard	--	--
	Phylpro	--	--

In addition to the large-scale recombination event, recombination signals were also detected within the spike (S) gene of PDCoV-ZJHZ2024 (Event No. 4). This event spanned alignment positions 130–1,828 of the S gene (130–1,823 after gap removal) and was identified by the MaxChi, Chimaera, and SiScan methods (Table 2). Although supported by fewer algorithms and showing lower statistical significance than the whole-genome event, MaxChi and SimPlot analyses still revealed local changes in sequence similarity between PDCoV-ZJHZ2024 and the putative parental strains within the corresponding region (Figures 4B,D and Supplementary Figure S4B). Consistently, region-specific phylogenetic analyses demonstrated a degree of topological incongruence among trees reconstructed from different S gene fragments (Supplementary Figure S3), suggesting a localized genetic rearrangement within the S gene, albeit with weaker supporting evidence.

**Figure 5:**
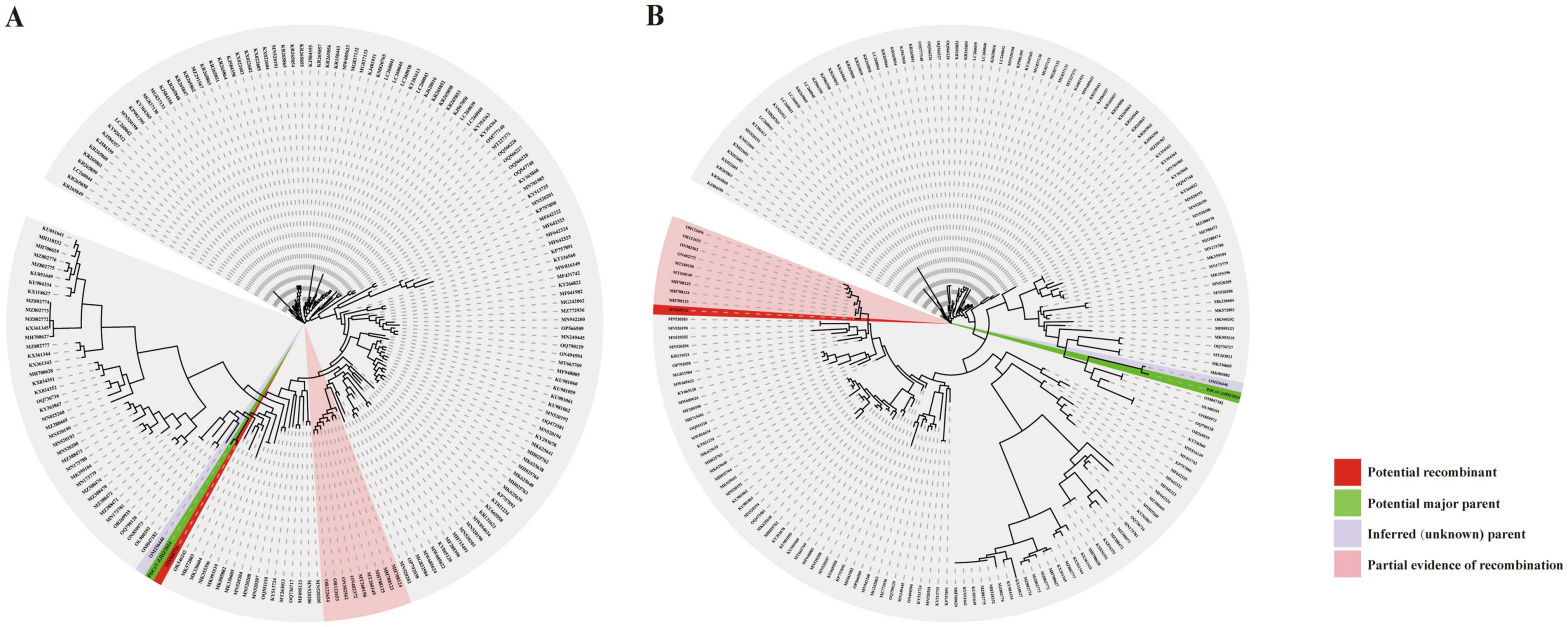
Phylogenetic analyses of PDCoV based on non-recombinant and recombinant genomic regions. **(A)** Whole-genome phylogenetic tree reconstructed using the non-recombinant regions. The non-recombinant regions were defined as genomic segments outside the inferred recombination breakpoints, corresponding to alignment positions 1–18,957 and 20,799–25,422 (positions 1–18,940 and 20,779–25,422 after gap removal). In this tree, PDCoV-ZJHZ2024 (highlighted in red) clusters with the putative minor parental lineage, supporting its genetic origin in the non-recombinant regions. **(B)** Phylogenetic tree reconstructed using the recombinant region. The recombinant fragment spans alignment positions 18,958–20,798 (positions 18,941–20,778 after gap removal). The 99% confidence intervals (CI) for the start and end breakpoints are 18,582–19,198/18,565–19,181 and 20,537–20,892/20,517–20,872, respectively (alignment positions/gap-removed positions). In this tree, PDCoV-ZJHZ2024 clusters with the putative major parental lineage, exhibiting a phylogenetic placement distinct from that observed in the non-recombinant regions. In both trees, red branches indicate the putative recombinant strain (ON968724), green branches represent the putative major parent (PDCoV-ZJHZ2024), purple branches denote inferred but unclassified parental lineages, and pink shaded sectors indicate phylogenetic regions providing supportive evidence for recombination. Together, these phylogenetic patterns are consistent with the recombination detection results and further validate the mosaic genomic structure of PDCoV-ZJHZ2024.

To further validate the parental origins of the detected recombination events, phylogenetic trees were reconstructed separately based on recombinant and non-recombinant genomic regions. In trees inferred from the non-recombinant regions (alignment positions 1–18,957 and 20,799–25,422), PDCoV-ZJHZ2024 clustered with the inferred minor parental lineage, whereas reconstruction based on the recombinant fragment (positions 18,958–20,798) placed PDCoV-ZJHZ2024 within the major parental clade (Figures 5A,B). The pronounced topological incongruence between different genomic regions represents a hallmark of recombination-driven evolution and further supports the mosaic genomic structure of PDCoV-ZJHZ2024. Notably, phylogenetic analysis of the ORF1b region showed that PDCoV-ZJHZ2024 retained a conserved evolutionary position consistent with the non-recombinant portions of the genome (Supplementary Figure S4D), indicating that ORF1b was not involved in the major recombination event and may serve as a reliable reference for defining recombination boundaries. Collectively, these findings demonstrate that whole-genome-scale recombination constitutes the primary evolutionary mechanism shaping PDCoV-ZJHZ2024, whereas the recombination signal detected within the S gene likely represents a secondary or localized event.

## Discussion

In recent years, the incidence of viral co-infections in swine populations has risen markedly, particularly in diarrheal diseases where PEDV–PDCoV co-infection is most frequently reported (38–42). Co-infections involving PCV and either PEDV or PDCoV have also been documented. When multiple viruses exploit the same host receptors—such as ACE2, APN, or DPP4—competitive binding may occur, reducing entry efficiency. Viral infections can further upregulate or redistribute host receptors, facilitating subsequent pathogen entry (43). In this study, although PCV-related sequences were detected, the BLAST alignment coverage was only approximately 13%, suggesting that these sequences more likely represent conserved fragments of PCV2 or PCV-like sequences rather than a complete PCV2 genome. Therefore, the available evidence is insufficient to support the presence of an active PCV2 infection in the samples, and the interpretation of viral co-infection should be made with caution. In some cases, one virus may exploit membrane remodeling or fusion pores induced by another, enabling entry through non-canonical pathways even without direct receptor recognition (44). Beyond changes in entry, co-infections create a favorable environment for recombination and adaptive evolution (45). Mutations in functional domains—especially the spike (S) protein—can alter receptor affinity, broaden host range, and enhance infectivity. In PDCoV–SADS-CoV co-infection, these viruses may compete for APN binding or modulate receptors in antagonistic or synergistic ways (46, 47).

Geographic variation acts as another external driver of recombination (48). Differences in viral lineages, host population structures, and farming practices enable genetically distinct strains to co-infect single hosts. Cross-regional pig transport mixes divergent viral populations, raising recombination risk. In regions with weak biosecurity, the co-circulation of multiple strains fosters novel recombinants. In this study, phylogenetic incongruence between the S gene and the complete genome of PDCoV-ZJHZ2024 suggested recombination, possibly reflecting cross-regional transmission under insufficient biosecurity. Multiple nonsynonymous S gene mutations indicate adaptive evolution under both recombination and natural selection. Notably, pigs serve as “mixing vessels” for coronaviruses, as they express multiple receptors (APN, ACE2, DPP4), elevating cross-species transmission risk and underscoring public health implications.

Latent infections and low-level viral persistence are also important in co-infection contexts (49). Viruses such as SADS-CoV can persist at low titers with mild or non-specific symptoms, escaping routine detection. These latent states enable long-term maintenance and may reactivate under stress or secondary infections, sparking outbreaks. Although we did not obtain a complete SADS-CoV genome, ORF1a and S gene fragments detected by RT-PCR indicate low-level infection. Such infections facilitate silent transmission and provide a temporal window for recombination and adaptive mutations. Even low-level SADS-CoV expression during co-infection could permit template switching with PDCoV, particularly in the S gene. Prolonged persistence further allows gradual accumulation of adaptive mutations under incomplete immune clearance, enhancing receptor binding or immune evasion. These mechanisms contribute to persistence and increase cross-species risk. Surveillance must therefore consider latent phases and low viral loads. Integrating high-sensitivity RT-PCR with broad-range sequencing will improve detection of covert infections. Continuous monitoring of subclinically infected animals is essential for understanding transmission dynamics and the role of latency in recombination and evolution.

Microbiota dysbiosis also contributes to pathogenesis (50, 51). Coronaviruses can disrupt gut homeostasis via epithelial damage and inflammation, promoting opportunistic pathogens. In this study, PDCoV likely drove microbiota disruption. A recent study showed PDCoV infection reduced *Bacteroides fragilis* abundance, impairing bile acid metabolism. This led to accumulation of conjugated bile acids and reduced lithocholic acid (LCA), a secondary bile acid that blocks PDCoV entry by disrupting S–APN interactions. Restoring *B. fragilis* colonization or supplementing LCA suppressed PDCoV replication *in vitro* and *in vivo* (52). These findings reveal a microbiota-mediated antiviral mechanism and suggest probiotic or microbial restoration therapies as adjunct strategies.

In conclusion, viral co-infection, recombination-driven evolution, and microbiota dysbiosis form a complex network driving swine diarrheal diseases. Future research should clarify virus–virus and virus–host interactions, particularly in receptor modulation, membrane fusion, and immune evasion. The role of co-infections in cross-species transmission also deserves emphasis, given pigs’ status as key intermediate hosts. This study’s limitations—including the absence of a complete SADS-CoV genome and reliance on fragmentary detection—should be acknowledged, and larger-scale genomic–microbiome surveillance is warranted. From a preventive standpoint, broad-spectrum or multivalent vaccines should be prioritized, as recombination can undermine strain-specific protection. Finally, based on the virus–microbiota synergism hypothesis, future strategies should integrate microbiome-targeted interventions with conventional vaccines and biosecurity to safeguard herd health, curb viral evolution, and establish effective early-warning systems for major infectious diseases.

## Conclusion

A novel PDCoV strain, PDCoV-ZJHZ2024, was identified from diarrheic pigs in Zhejiang Province, China, in association with SADS-CoV co-infection and gut microbiota dysbiosis. Phylogenetic and recombination analyses revealed cross-regional genetic exchange within the ORF1b and spike (S) gene regions, underscoring the central role of recombination in PDCoV evolution. Codon usage analysis indicated natural selection as the dominant force shaping codon bias, potentially optimizing host translational efficiency. These findings broaden the understanding of PDCoV diversity and evolution and provide a scientific basis for strengthening surveillance and guiding preventive strategies in swine populations.

## Data Availability

The complete viral genome sequences identified in this study were deposited in GenBank under the accession numbers PX492333.
