# Supplementary material for: Molecular characterization and evolutionary dynamics of a recombinant PDCoV strain in a swine diarrhea epidemic with SADS-CoV co-infection
Source: Front Vet Sci. 2026 Feb 18;13:1749819. doi: 10.3389/fvets.2026.1749819 (PMC12956720; doi:10.3389/fvets.2026.1749819)
Supplement: Supplementary file 1 [file Data_Sheet_1.docx]

Supplementary Materials

**Supplementary Figures:**

**
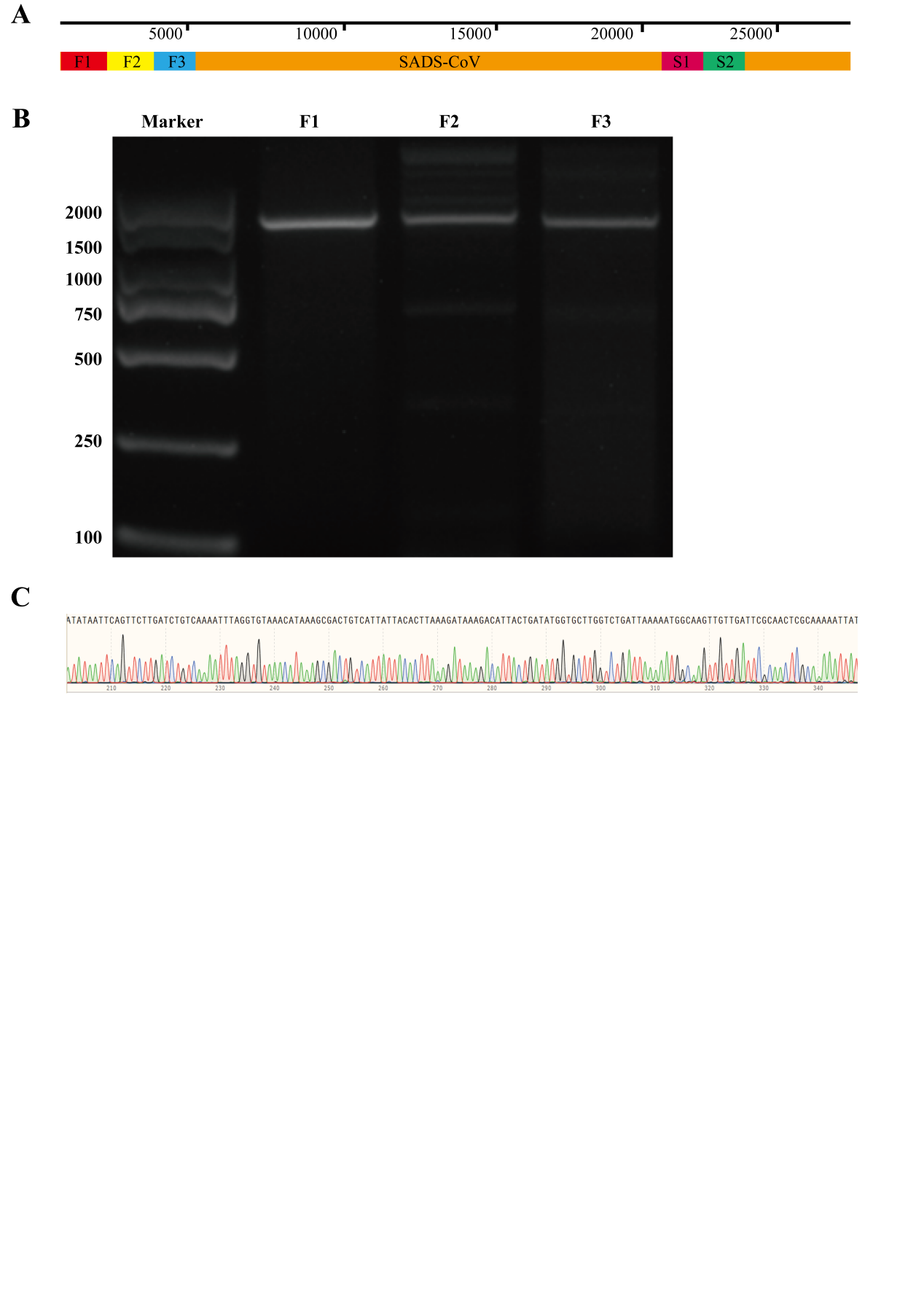
**

**Figure S1. Amplification and sequence verification of SADS-CoV genomic fragments.** (A) Schematic diagram of the SADS-CoV genome and primer locations. (B) Agarose gel electrophoresis of PCR products corresponding to fragments F1, F2, and F3. (C) Representative Sanger sequencing chromatogram of a PCR product from the S1 region of the spike (S) gene.


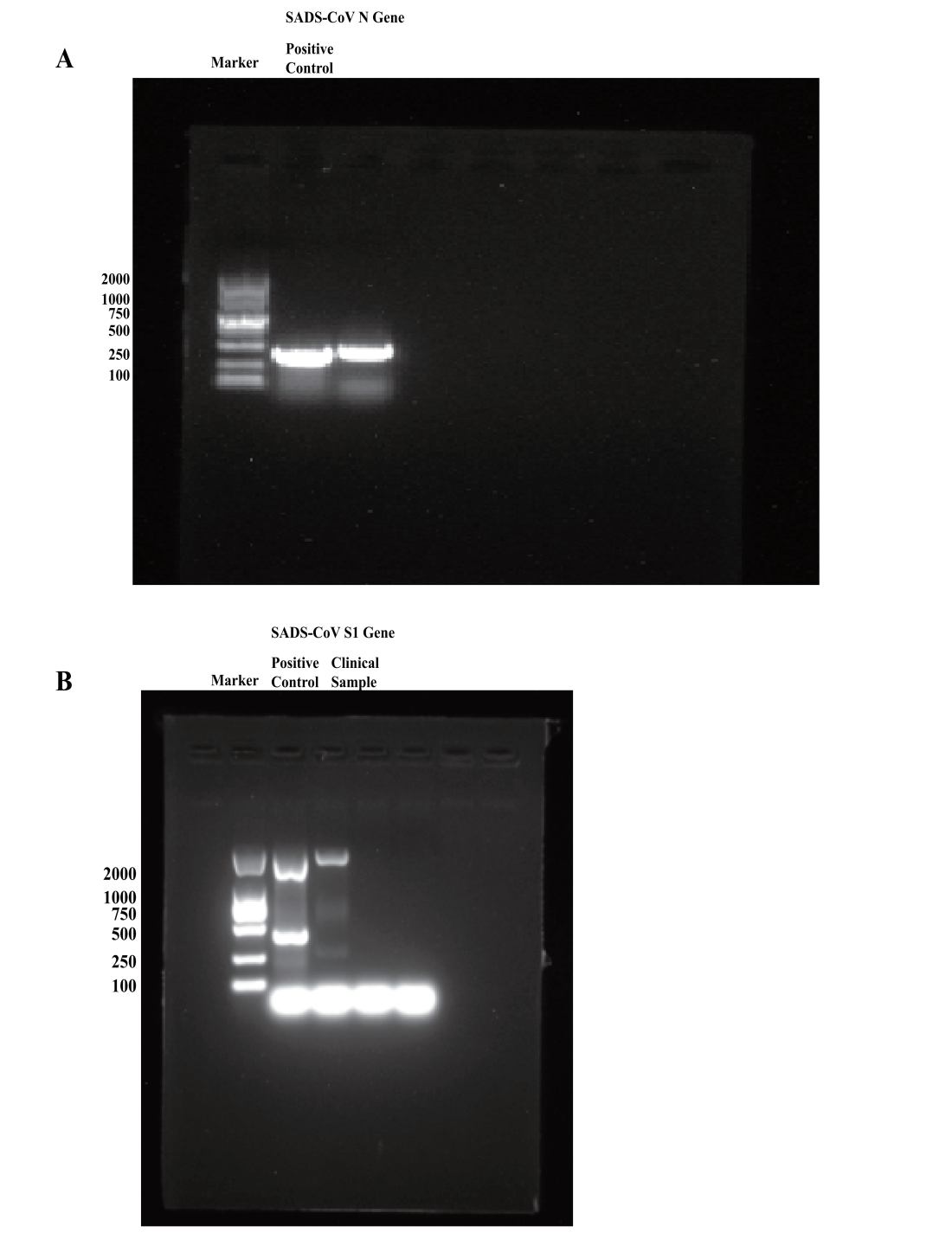


**Figure S2. Detection of SADS-CoV N and S1 genes in clinical samples (unprocessed original images).** (A) RT-PCR detection of the SADS-CoV nucleocapsid (N) gene by agarose gel electrophoresis. (B) RT-PCR detection of the SADS-CoV spike S1 gene by agarose gel electrophoresis.


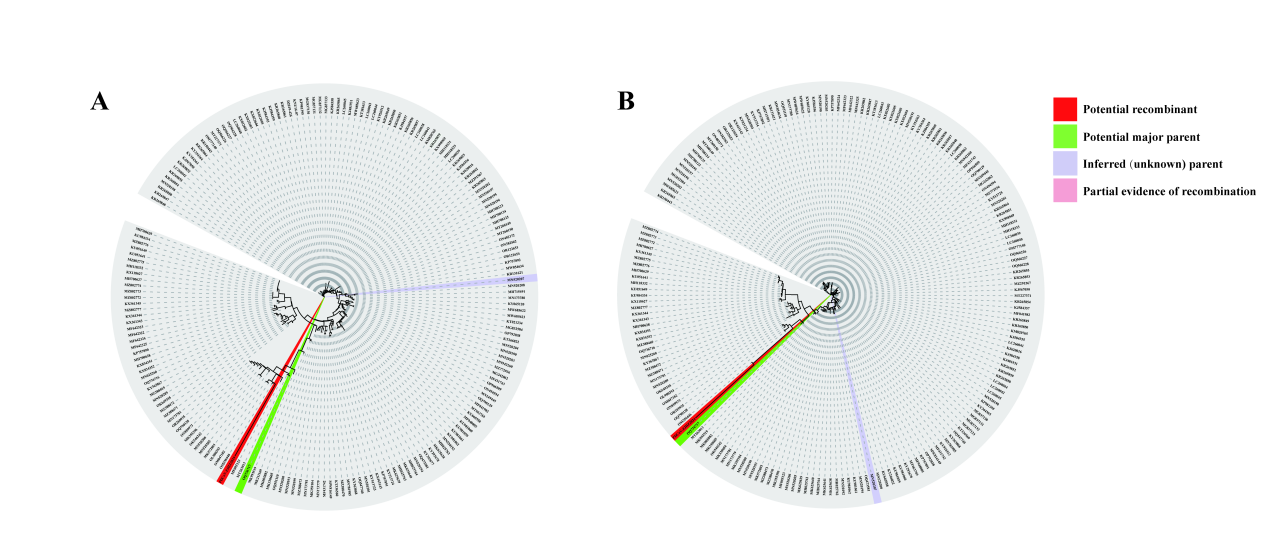


**Figure S3. Phylogenetic evidence for recombination in the spike (S) gene of PDCoV-ZJHZ2024.** (A) Tree constructed using the non-recombinant regions of the S gene. (B) Tree constructed using the recombinant region of the S gene.

**Figure S4. Recombination analyses and supporting phylogenetic evidence for PDCoV-ZJHZ2024.** (A) Whole-genome MaxChi breakpoint matrix. (B) MaxChi breakpoint matrix based on the S gene. (C) BootScan analysis (bootstrap support), illustrating a clear shift in phylogenetic clustering between the major and minor parental strains across the recombinant region. （D）Maximum-likelihood phylogenetic tree based on ORF1b gene sequences of representative PDCoV strains. PDCoV-ZJHZ2024 is indicated by a red star.

**Table S1. Virus RT-PCR detection primer information**

| **Virus** | **Primer Names** | **Primer Sequence( 5' to 3' )** |
| --- | --- | --- |
| SADS-CoV | SADS-RT-F | TGAACAGGCGGAATCTCGTGG |
|  | SADS-RT-R | ACTGCGGCTTTGACTTTGATT |
| TGEV | TGEV-RT-F | GGTCGGAAGAATAATAACATAC |
|  | TGEV-RT-R | TTGGATTGTTGCCTGCCTCTA |
| PEDV | PEDV-RT-F | GCAAACGGGTGCCATTATCTC |
|  | PEDV-RT-R | ATTGTTGCCATTGCCACGACT |
| PDCoV | PDCoV-RT-F | CCCAACAATCCTAAACATCAG |
|  | PDCoV-RT-R | GAACCCTCCTTGACTGTGATT |

| **ORF1ab** | | | |
| --- | --- | --- | --- |
| **Codon position** | **Codon change** | **Corresponding amino**  **acid change** | **Corresponding protein change** |
| 760 | G to T | Synonymous | ORF1ab |
| 1933 | T to C | Synonymous | ORF1ab |
| 5227 | A to G | T to A | ORF1ab |
| 6907 | T to C | Y to H | ORF1ab |
| 6921 | C to T | Synonymous | ORF1ab |
| 7471 | C to A | L to I | ORF1ab |
| 7729 | T to C | Y to H | ORF1ab |
| 8512 | C to T | R to W | ORF1ab |
| 10180 | A to G | S to G | ORF1ab |
| 10440 | A to G | Synonymous | ORF1ab |
| 10869 | A to C | E to D | ORF1ab |
| 11215 | C to T | P to S | ORF1ab |
| 12072 | A to G | Synonymous | ORF1ab |
| 18975 | T to C | Synonymous | ORF1ab |
| 19026 | C to T | Synonymous | ORF1ab |
| 19041 | C to T | Synonymous | ORF1ab |
| 19113 | T to A | Synonymous | ORF1ab |
| 19242 | T to G | Synonymous | ORF1ab |
| **Spike (S)** | | | |
| 19448 | A to C | D to A | S |
| 19480 | A to G | I to V | S |
| 19485 | C to T | Synonymous | S |
| 19502 | T to C | Synonymous | S |
| 19553 | C to T | Synonymous | S |
| 19604 | T to C | S to P | S |

**Table S2. Summary of nucleotide and amino acid mutations identified in the PDCoV-ZJHZ2024 genome.**

| 19627 | T to C | F to S | S |
| --- | --- | --- | --- |
| 19677 | G to A | Synonymous | S |
| 19703 | T to C | Synonymous | S |
| 19751 | C to T | H to Y | S |
| 19753 | T to C | H to Y | S |
| 19756 | C to T | Synonymous | S |
| 19792 | G to A | V to M | S |
| 19850 | T to C | Synonymous | S |
| 19856 | C to T | Synonymous | S |
| 19859 | T to C | Synonymous | S |
| 19866 | T to C | Synonymous | S |
| 19892 | C to T | H to Y | S |
| 19958 | T to G | V to G | S |
| 19964 | C to T | S to F | S |
| 20003 | C to T | Synonymous | S |
| 20132 | A to G | Synonymous | S |
| 20144 | C to T | Synonymous | S |
| 20171 | T to C | Synonymous | S |
| 20196 | C to T | M to T | S |
| 20237 | C to T | S to L | S |
| 20255 | C to T | P to L | S |
| 20274 | T to C | Synonymous | S |
| 20322 | A to G | Synonymous | S |
| 20369 | C to T | S to L | S |
| 20385 | T to C | I to T | S |
| 20426 | T to C | L to P | S |
| 20477 | C to T | S to F | S |
| 20576 | A to G | K to R | S |
| 20783 | C to T | S to F | S |
| 20786 | G to A | R to K | S |

| 20828 | C to T | T to I | S |
| --- | --- | --- | --- |
| 20838 | C to T | S to L | S |
| 20998 | T to C | L to R | S |
| 20999 | T to G | L to R | S |
| 21022 | C to T | L to F | S |
| 21033 | T to C | L to P | S |
| 21047 | T to C | M to T | S |
| 22151 | A to G | Y to C | S |
| 22451 | T to G | L to R | S |
| 22538 | T to C | F to S | S |
| 22572 | G to C | L to F | S |
| 20838 | C to T | S to L | S |
| 20998 | T to C | L to R | S |
| 20999 | T to G | L to R | S |
| 21022 | C to T | L to F | S |
| 21033 | T to C | L to P | S |
| 21047 | T to C | M to T | S |
| 22151 | A to G | Y to C | S |
| 22451 | T to G | L to R | S |
| 22538 | T to C | F to S | S |
| 22572 | G to C | L to F | S |
| 20838 | C to T | S to L | S |
| 20998 | T to C | L to R | S |
| 20999 | T to G | L to R | S |
| 21022 | C to T | L to F | S |
| 21033 | T to C | L to P | S |
| 21047 | T to C | M to T | S |
| 22151 | A to G | Y to C | S |
| 22451 | T to G | L to R | S |
| 22538 | T to C | F to S | S |
| 22572 | G to C | L to F | S |

| 20998 | T to C | L to R | S |
| --- | --- | --- | --- |
| 20999 | T to G | L to R | S |
| 21022 | C to T | L to F | S |
| 21033 | T to C | L to P | S |
| 21047 | T to C | M to T | S |
| 22151 | A to G | Y to C | S |
| 22451 | T to G | L to R | S |
| 22538 | T to C | F to S | S |
| 22572 | G to C | L to F | S |
| **M** | | | |
| 23484 | A to G | Synonymous | M |
| 23516 | A to C | E to A | M |
| **NS6** | | | |
| 23768 | C to T | T to I | NS6 |
| **5′ and 3′ UTRs** | | | |
| 31-39 | (CTACCGACA) missing | (LPT) Missing | 5' UTR |
| 25433-25435 | CCT missing | K missing | 3' UTR |
| 25438-25440 | GAC missing | K missing | 3' UTR |
| 25451 | A to C | Not applicable | 3' UTR |

**Table S3. Confidence analysis of recombination events in PDCoV whole genome and S gene.**

| **Recombination**  **Event Number** | **Methods** | **#seqs detected in** | **Av. P-Val** |
| --- | --- | --- | --- |
| Confidence Analysis of Whole-Genome Recombination Event 16 in PDCoV | RDP | -- | -- |
|  | GENECONV | 10 | 2.771×10^-3^ |
|  | BootScan | 4 | 1.577×10^-6^ |
|  | MaxChi | 10 | 2.011×10^-5^ |
|  | Chimaera | 10 | 7.816×10^-5^ |
|  | SiScan | 3 | 7.899×10^-9^ |
|  | 3Seq | 1 | 3.790×10^-11^ |
|  | Lard | -- | -- |
|  | Phylpro | -- | -- |
| Confidence Analysis of S-Genome Recombination Event 4 in PDCoV | RDP | -- | -- |
|  | GENECONV | -- | -- |
|  | BootScan | -- | -- |
|  | MaxChi | 1 | 4.030×10^-3^ |
|  | Chimaera | 1 | 3.148×10^-2^ |
|  | SiScan | 1 | 2.910×10^-5^ |
|  | 3Seq | -- | -- |
|  | Lard | -- | -- |
|  | Phylpro | -- | -- |

**Table S4. Recombination signal analysis in the S gene of PDCoV.**

| **Event**  **number**  **(No.)** | **Found in** | **Recomb.** | **Major parent** | **Minor parent** | **Detection methods** | | | | | | |
| --- | --- | --- | --- | --- | --- | --- | --- | --- | --- | --- | --- |
|  |  |  |  |  | **R** | **G** | **B** | **M** | **C** | **S** | **T** |
| 1 | 1 | KT021234 | OK649355 | MZ388470 | **-** | **+** | **-** | **-** | **-** | **-** | **-** |
| 2 | 1 | ON968724 | PDCoV-ZJHZ2024 | MF948005 | **-** | **-** | **-** | **-** | **-** | **-** | **+** |
| 3 | 4 | MZ388470 | MN520198 | OK649355 | **-** | **+** | **-** | **-** | **-** | **+** | **-** |
| **4** | **1** | **PDCoV-ZJHZ2024** | **OQ736717** | **MN520207** | **-** | **-** | **-** | **+** | **+** | **+** | **-** |
| 5 | 9 | KX443143 | MN520198 | MN781985 | **-** | **+** | **-** | **-** | **-** | **-** | **-** |

*Algorithms Used for Recombination Detection: RDP(R), GENECONV(G), BootScan(B), MaxChi(M), Chimaera(C), SiScan(S),TOPAL DSS(T).
